# Genomics: Infectious Disease and Host–Pathogen Interaction

**DOI:** 10.3390/ijms24021748

**Published:** 2023-01-16

**Authors:** Franklin Wang-Ngai Chow

**Affiliations:** Department of Health Technology and Informatics, The Hong Kong Polytechnic University, Hunghom, Hong Kong, China; franklin.chow@polyu.edu.hk

## 1. Introduction

Infectious diseases, which are caused by pathogens such as bacteria, viruses, fungi, and parasites, pose a serious threat to humans, animals, and plants. In order to establish, spread, and promote infections, pathogens can interact with their hosts to inhibit or escape the host immune system. Unique genomic features, such as mutations or species-specific genes, significantly influence the outcomes of host–pathogen interactions, including virulence, resistance phenotypes, and disease severity. With the development of NGS and nanopore sequencing, genomic, transcriptomic, metagenomic, and small RNA research is now extremely cost-effective. Recent research has demonstrated that host–pathogen interactions can be bi-directional through both virulence genes from pathogens, and hosts’ own defense mechanisms. Aside from this, the development of viral detection methods based on the study of phylogenomics and mutations of viral genomes is always beneficial to the microbiology field.

This Special Issue of the *International Journal of Molecular Sciences* entitled “Genomics: Infectious Disease and Host –Pathogen Interaction” contains twelve original articles and three reviews that provide new insights into infectious diseases with regard to biofilm formation [1,2,3], diagnostics [4], vaccine development [5], and host–pathogen interaction [6,7]; these insights are based on genomic [8,9,10,11,12], transcriptomic [13,14], and microbiomic [15] studies.

## 2. Bacteria and Biofilm Formation

Biofilm formation is a process in which microorganisms attach to and grow on a surface, forming an extracellular matrix that facilitates adhesion, resulting in alterations in phenotypes in terms of growth rate, gene transcription, and antibiotic resistance. Jahan and co-workers reviewed the complex mechanism of biofilm formation and its link to the pathogenicity of the bacterium *Salmonella typhi*, the causative agent of typhoid fever [3]. In another study, Shenkutie et al. found that biofilm-specific antibiotic resistance and the virulence genes of *Acinetobacter baumannii* can be induced by sub-minimum inhibitory concentrations of imipenem and colistin, leading to higher antibiotic tolerance in biofilm formation [1]. On the other hand, unexpectedly, the recombinant SARS-CoV-2 spike protein and mouse coronavirus are able to inhibit biofilm formation of common bacterial pathogens such as *Streptococcus pneumoniae* and *Staphylococcus aureus*. This suggests the potential of a more virulent planktonic lifestyle of these commensal bacteria in secondary pneumonia in COVID-19 patients [2]. Aside from biofilm studies, a comprehensive study by Rai N et al. focuses on understanding the formation and mechanisms of the anticipatory response in *Escherichia coli*. The authors performed whole genome random mutagenesis and identified the fitness of *E. coli* strains with positive association in the d-galactose treatment and expression of the maltose operon, which were found to be lower than that of the wild-type strain by up to 20% [11]. 

## 3. Diagnostics

Pathogen detection assays and diagnostic techniques have advanced further in recent years. For example, FTA cards and related products simplify the collection, transportation, and storage of biological sample fluids and are an easy and cost-effective option for viral diagnostics. Elnagar et al. found that FTA cards are highly effective in releasing nucleic acids of African swine fever virus (ASFV) and influenza A virus (IAV) for subsequent diagnostic procedures [4]. While molecular detection assays are vital for infectious disease diagnosis, the immunization of high-risk groups to prevent infectious illnesses is equally important. There is a novel vaccination strategy that uses engineered trypanosomes expressing a PrP-specific peptide to induce a strong humoral immune response that protects immunized mice from the prion disease transmissible spongiform encephalopathies [5].

## 4. Genomics and Transcriptomics on Host–Pathogen Interactions

Genomics and transcriptomic analysis are involved in the investigation of infectious diseases and host–pathogen interactions as well. While innate immune responses such as type I interferon (IFN) family cytokines can be stimulated by viral infections, Barik S. reviewed the mechanisms whereby IFN further triggers a STAT2 signaling cascade leading to an antiviral state of the cell [7]. On the other hand, the mapping of small RNA reads from Huh7 cells infected with Ebola virus to the Ebola genome revealed the presence of two novel Ebola-virus-derived microRNAs which may target host genes [13]. Serial passaging of seasonal H3N2 influenza A virus in host cells was able to induce mutations across different ORFs, driving the viral genome to evolve and perhaps altering viral virulence [12]. In porcine, although the newly identified porcine circovirus (PCV) PCV4 is prevalent in pigs, the genomes of PCV4 are relatively stable among the circulating strains. Further genomic analysis indicates that PCV4 is more similar to mink circovirus (MiCV), suggesting that PCV4 may have been derived from MiCV or have a common ancestor with MiCV, or that mink is an intermediate host of PCV4 [9]. 

In plants, plant viruses can hijack host systems for their own needs; in some cases, symptoms of plant diseases have been linked with virus colonization of the xylem and some host genes interact with the virus in the plant’s xylem tissue [6]. Other than viruses, stripe rust which is caused by the fungus *Puccinia striiformis* f. sp. *tritici* (*Pst*) is a destructive disease to wheat worldwide. Nineteen SNPs based on secreted proteins were found to have significant associations with twelve avirulence genes using a transcriptomics-based approach, providing prospective insights into avirulence genes and host–pathogen interactions [10]. To facilitate the study of the plant–pathogen interaction, a database of the forage crops, Alfalfa, and the emerging bacterial stem blight, *Pseudomonas syringae* pv. *syringae* ALF3, was set up. This database contains *Pseudomonas* proteome annotations and host–pathogen interactome tools that predict the interactions between hosts and pathogens based on orthology [8]. 

In parasitic protozoan, a less virulent strain of *Leishmania infantum* (BOS1FL1) isolated from a canine outbreak in Spain was compared to the reference strain (BCN150) with a canine macrophage infection model by using a transcriptomic approach. Surprisingly, pathways such as phagocytosis and the signal transduction of TNF, MAK, and NFκB were only altered after infection with BOS1FL1 but not with the reference strain, suggesting that low virulence of the BOS1FL1 strain may be due to these mechanisms [14]. 

## 5. Microbiomics

Microbiomics is the study of microbiomes that exist in a particular environment, and uses multi-omics approaches. Jo Y et al. compared two distinct library types (DNA for metagenomics, RNA for metatranscriptomics) and three different analytical methods for the identification of microbiome in overwintering pepper fruits. Their results revealed that DNA sequencing is more useful for the identification of bacteria and DNA viruses, whereas mRNA sequencing is superior for identifying fungi and RNA viruses [15].

In summary, this Special Issue discusses previous and current research, as well as mechanisms and lines of thought that correlate with the genomics of infectious diseases, pathogens, and the host.
